# Altered glutamate–glutamine and amide proton transfer-weighted values in the hippocampus of patients with amnestic mild cognitive impairment: A novel combined imaging diagnostic marker

**DOI:** 10.3389/fnins.2023.1089300

**Published:** 2023-02-23

**Authors:** Xin Chen, Tao Gong, Tong Chen, Changyuan Xu, Yuchao Li, Qingxu Song, Liangjie Lin, Georg Oeltzschner, Richard A. E. Edden, Zhangyong Xia, Guangbin Wang

**Affiliations:** ^1^Department of Radiology, Shandong Provincial Hospital Affiliated to Shandong First Medical University, Jinan, China; ^2^Department of Neurology, Liaocheng People's Hospital, Liaocheng, China; ^3^Department of Radiology, Shandong Provincial Hospital, Cheeloo College of Medicine, Shandong University, Jinan, China; ^4^Department of Ultrasound, Shandong Provincial Hospital Affiliated to Shandong First Medical University, Jinan, China; ^5^Department of Radiation Oncology, Qilu Hospital of Shandong University, Jinan, China; ^6^Philips Healthcare, Beijing, China; ^7^The Russell H. Morgan Department of Radiology and Radiological Science, Johns Hopkins University School of Medicine, Baltimore, MD, United States; ^8^F.M. Kirby Research Center for Functional Brain Imaging, Kennedy Krieger Institute, Baltimore, MD, United States; ^9^Department of Neurology, Liaocheng Clinical School of Shandong First Medical University, Liaocheng, China

**Keywords:** amnestic mild cognitive impairment, hippocampus, MEGA-PRESS, amide proton transfer-weighted imaging, imaging diagnostic marker

## Abstract

**Background and purpose:**

Early diagnosis of amnestic mild cognitive impairment (aMCI) and timely management to delay the onset of Alzheimer's disease (AD) would benefit patients. Pathological metabolic changes of excitatory/inhibitory neurotransmitters and abnormal protein deposition in the hippocampus of aMCI may provide a new clue to imaging diagnosis. However, the diagnostic performance using these hippocampal metabolite measurements is still unclear. We aimed to quantify right hippocampal glutamate–glutamine (Glx) and gamma-aminobutyric acid (GABA) levels as well as protein-based amide proton transfer-weighted (APTw) signals of patients with aMCI and investigate the diagnostic performance of these metabolites.

**Methods:**

In this cross-sectional study, 20 patients with aMCI and 20 age- and gender-matched healthy controls (HCs) underwent MEGA Point Resolved Spectroscopy (MEGA-PRESS) and APTw MR imaging at 3 T. GABA+, Glx, and APTw signals were measured in the right hippocampus. The GABA+ levels, Glx levels, Glx/GABA+ ratios, and APTw values were compared between the HCs and aMCI groups using the Mann–Whitney U test. Binary logistic regression and receiver operating characteristic (ROC) curve analyses were used to evaluate MEGA-PRESS and APTw parameters' diagnostic performance.

**Results:**

Compared with HCs, patients with aMCI had significantly lower Glx levels in the right hippocampus (7.02 ± 1.41 i.u. vs. 5.81 ± 1.33 i.u., *P* = 0.018). No significant changes in the GABA+ levels were observed in patients with aMCI (HCs vs. aMCI: 2.54 ± 0.28 i.u. vs. 2.47 ± 0.36 i.u., *P* = 0.620). In addition, Glx/GABA+ ratios between the two groups (HCs vs. aMCI: 2.79 ± 0.60 vs. 2.37 ± 0.55, *P* = 0.035) were significantly different. Compared with HCs, patients with aMCI showed higher APTw values in the right hippocampus (0.99 ± 0.26% vs. 1.26% ± 0.28, *P* = 0.006). The ROC curve analysis showed that Glx, GABA+, Glx/GABA+, and APTw values had an area under the curve (AUC) of 0.72, 0.55, 0.70, and 0.75, respectively, for diagnosing aMCI. In the ROC curve analysis, the AUC of the combination of the parameters increased to 0.88, which is much higher than that observed in the univariate analysis (*P* < 0.05).

**Conclusion:**

The combination of right hippocampal Glx levels and APTw values improved the diagnostic performance for aMCI, indicating it as a promising combined imaging diagnostic marker. Our study provided a potential imaging diagnostic strategy of aMCI, which may promote early detection of aMCI and facilitate timely intervention to delay the pathological progress toward AD.

## Introduction

With the global population aging, Alzheimer's disease (AD) is an increasing public health concern, imposing a huge burden on families and society (Collaborators GBDDF, 2022). However, there is currently no cure for AD. Mild cognitive impairment (MCI) is considered to be a transitional stage between aging and AD (Petersen et al., 2018), and the overall prevalence of MCI was ~22% in the population aged 65 years and older (Manly et al., 2022). Amnestic mild cognitive impairment (aMCI) characterized by memory impairments is defined as a precursor to AD and is almost two times as likely to progress to dementia than non-amnestic (Glynn et al., 2021). Early diagnosis of aMCI and timely intervention to delay the onset of AD would be beneficial for patients (Levey et al., 2006).

Previous studies demonstrated that glutamatergic and/or γ-aminobutyric acidergic (GABAergic) dysfunction in excitatory and inhibitory (E/I) imbalance and consequent abnormal protein deposition contribute to the process of AD (Bi et al., 2020; Bukke et al., 2020; Conway, 2020; Jimenez-Balado and Eich, 2021), which emerges in MCI and preclinical stages of AD (Bell et al., 2007; Palop and Mucke, 2016). The hippocampus is a critical region for episodic memory and is highly vulnerable in the course of AD (Tulving and Markowitsch, 1998; Poulakis et al., 2018). Therefore, these abnormal hippocampal metabolites may be important clues for the early detection of MCI.

It is believed that glutamate (Glu) and GABA are two of the most important excitatory and inhibitory neurotransmitters in the brain and play critical roles in regulating and synchronizing neuronal signaling in the hippocampus (Kullmann and Semyanov, 2002; Sun et al., 2009). With magnetic resonance spectroscopy (MRS), glutamate–glutamine (Glx) and GABA levels can be measured non-invasively. Edited MRS, such as Mescher–Garwood point resolved spectroscopy (MEGA-PRESS), provides selective detection of GABA and Glx (Mescher et al., 1998; Harris et al., 2017), and has been applied in patients with neuropsychiatric disorders (Edden et al., 2012; Cao et al., 2018). Recently, hippocampal Glx/GABA levels in patients with MCI have been explored using MRS in a few clinical studies, with diverse results: there is reduced glutamate in the left hippocampus (Wong et al., 2020) but no visible changes of glutamate in the right hippocampus (Rupsingh et al., 2011; Huang et al., 2017); in addition, no significant alteration of the GABA levels has been observed in the right hippocampus (Huang et al., 2017). The hippocampus is adjacent to the bone and the sinuses, causing heterogeneity of the B0 magnetic field and extensive susceptibility variations. Moreover, the hippocampus is small, especially in older patients with AD and is located deep in the temporal lobe, resulting in a poor signal-to-noise ratio. Although the hippocampus is a vital and vulnerable region in AD, it is still challenging to acquire the edited MRS in this region (Hsu et al., 2001). Abnormal accumulations of amyloid beta and tau protein are hallmark pathological alterations of the MCI and AD brain, especially in the hippocampal region (Mufson et al., 2012). With amide proton transfer-weighted (APTw) imaging, a chemical exchange saturation transfer (CEST)-based molecular MRI technique, low-concentration endogenous proteins and peptides in tissues can be detected (Zhou et al., 2003). A recent study has shown that the APTw values of the bilateral hippocampus in patients with AD were significantly higher than those in healthy elders (Wang et al., 2015). APTw imaging is a potential method to visualize the abnormal proteins of patients with aMCI, but the diagnostic performance of hippocampal APTw is still unsatisfactory (Zhang et al., 2020).

Aberrant hippocampal Glx/GABA levels and APTw values may arise in patients with aMCI, which may be a promising clue of imaging diagnosis. However, the combined diagnostic performance for MCI using these hippocampal metabolite measurements is still unclear. We hypothesized that the combination of Glx/GABA levels and APTw values might be a promising imaging diagnostic strategy for MCI. Therefore, in this study, MEGA-PRESS and APTw imaging were used to test for differences in the Glx/GABA levels and APTw signals in the hippocampus of patients with aMCI and further investigate the combined diagnostic performance of these markers.

## Materials and methods

### Participants

This study was ethically approved by the institutional review board at Shandong Provincial Hospital, and all participants provided written informed consent. Patients with aMCI enrolled in this study met the following criteria: (1) between 50 and 80 years of age; (2) fulfilling the clinical features proposed by the 2011 revised National Institute on Aging–Alzheimer's Association (NIA-AA) criteria for MCI (Albert et al., 2011) and current patients with aMCI (MCI with memory impairments); and (3) having a score of 20 or higher on the Minimum Mental State Examination (MMSE) for elementary school and >24 in the middle school and above group, with Clinical Dementia Rating (CDR) of 0.5. The exclusion criteria for participants included: (1) seizure disorders; (2) serious mental illness; (3) serious cerebrovascular diseases, traumatic brain injury, hydrocephalus, brain tumor, and white matter lesions (Fazekas ≥3); (4) alcohol or drug dependence; and (5) patients with MRI contraindications. The healthy controls were evaluated using the MMSE and CDR, and none of the controls had noticeable cognitive impairment. The diagnosis of aMCI was made by a neurologist with 8 years of experience. The MMSE and CDR evaluations were performed by a neuropsychologist with 5 years of experience blinded to the clinical information of all the participants. From December 2020 to March 2022, 20 patients with aMCI from the neurology outpatient clinic of Shandong Provincial Hospital in Jinan were recruited in our study. Twenty age- and gender-matched healthy controls (HCs) were recruited from the communities in Jinan. The patients' screening information is presented in the Supplementary Figure 1. All participants were right-handed. This is a cross-sectional study that followed the STROBE Guideline.

### MRI scanning protocol

**Figure 1:**
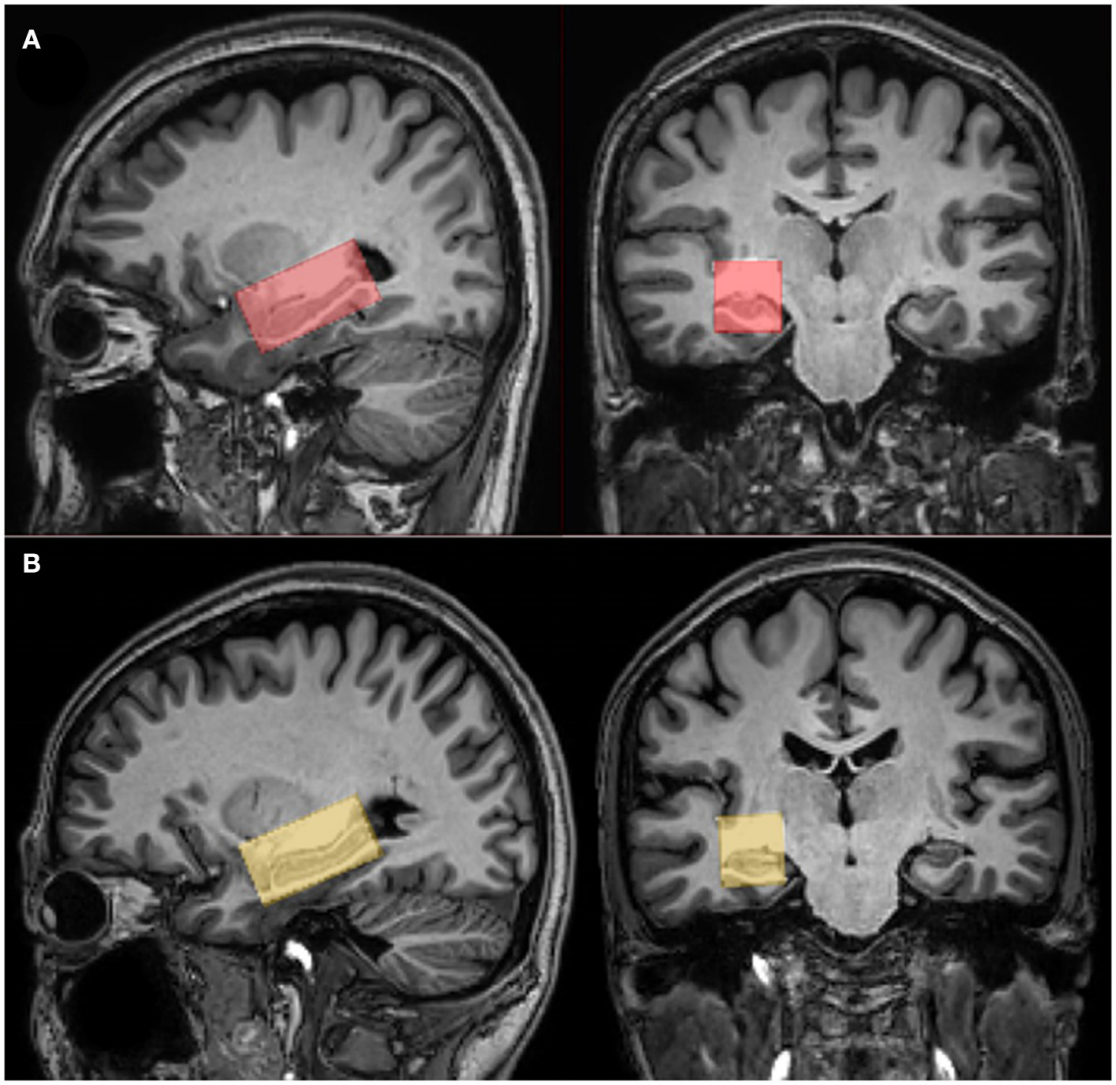
The position of voxels and segmentation data. Coronal and sagittal T1-weighted TFE images show single-voxel placements centered on the right hippocampus in a healthy participant [female, 64 years **(A)**] and a patient with amnestic mild cognitive impairment (aMCI) [women, 66 years **(B)**].

All participants were scanned using a 32-channel phased-array head coil with a 3.0 T scanner (Ingenia 3.0 CX; Philips Healthcare, Best, The Netherlands). The T1-weighted 3D turbo field echo sequence was acquired with the parameters as follows: repetition time/echo time (TR/TE) = 8.1/3.7 ms, slice thickness = 1 mm, field of view = 24 × 24 cm^2^, and voxel size = 1 × 1 × 1 mm^3^. The volume of interest (VOI) for MRS was centered on the right hippocampus and positioned parallel to the long axis of the hippocampal body in a parasagittal section with a size of 40 × 20 × 20 mm^3^ (Figure 1). The right hippocampus was chosen because the impairment of the right hippocampus is more notable than the left hippocampus in patients with AD (Geroldi et al., 2000; Barnes et al., 2005). In addition, measuring MRS of the unilateral hippocampus but not bilateral ones can shorten the examination time. GABA+ and Glx were detected using the MEGA-PRESS editing sequence, with the following parameters: T*R* = 2 s, TE = 68 ms, acquisition bandwidth = 2,000 Hz, and 160 averages. Before each acquisition, the voxels were automatically shimmered with FASTMAP. APTw imaging employed a 3D fast spin-echo sequence to collect data at seven saturation frequency points (± 2.7, ± 3.5, ± 4.3, and −1540 ppm) for the reconstruction of the Z-spectrum at every image voxel. B_0_ field map generation was performed three times using a saturation frequency of + 3.5 ppm and shifted echo times. Saturation radio frequency pulses for APTw imaging were implemented with an amplitude of 2 μT and a duration of 2 s. APTw sequence was also parallel to the hippocampal long axis to be positioned. The other parameters of 3D APTw imaging were as follows: T*R* = 6491 ms, TE = 8.3 ms, FOV = 230 × 180 × 60 mm^3^, reconstruction matrix = 256 × 256, reconstruction voxel size = 1.8 × 1.8 × 6 mm^3^, TSE facto*r* = 174. B_0_-corrected APTw images were reconstructed online after data collection.

### MRI data processing

Mescher–Garwood point resolved spectroscopy (MEGA-PRESS) data were analyzed using GANNET 3.0 (http://www.gabamrs.com/) in Matlab 2020b (Mathworks) (Edden et al., 2014). Moreover, macromolecules (MM) and homocarnosine (Rothman et al., 1997) contribute to the GABA signal at 3 ppm; therefore, it is referred to as GABA+ rather than GABA. Since the glutamine (Gln) signal was not separated from the glutamine (Glu) signal, we reported the Glx (the combined signals of Glu and Gln) level in our study. The ratios of the integrals of neurotransmitters (GABA+ or Glx) and water signals, corrected with T_1_/T_2_ relaxation and tissue composition, were used to calculate water-scaled GABA+ or Glx levels in institutional units (IUs) (Mullins et al., 2014; Harris et al., 2015).

Based on 3D T1-weighted brain images, the fractional gray matter (GM), white matter (WM), and cerebrospinal fluid (CSF) content within each spectroscopic voxel were calculated using an automatic brain segmentation program, FAST (FMRIB's automated segmentation tool) in the FSL package (Oxford University, Oxford, UK) (Zhang et al., 2001).

**Figure 2:**
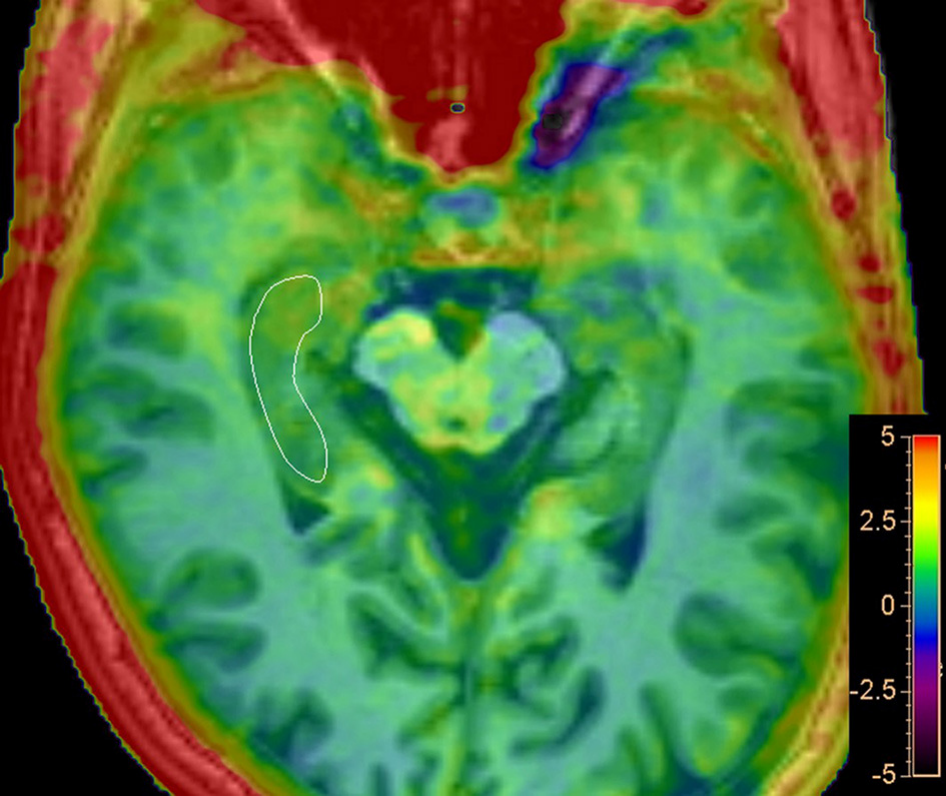
Representative regions of interest (ROIs) for APT analysis. Delineate ROI of the right hippocampus in the maximum cross-sectional layer of T_1_-weighted image with APTw image overlay.

Amide proton transfer-weighted (APTw) images were generated directly from the scanner, and they were co-registered and overlaid with geometrically identically acquired 3D T1WI images on a dedicated workstation called “IntelliSpace Portal” (Philips Healthcare, Best, the Netherlands) (Figure 2). The regions of interest (ROIs) were manually drawn on the fused image by two radiologists with 10 years of experience in neurological imaging blinded to clinical and cognitive information of all participants, to delineate the segments of the right hippocampus in the maximum cross-sectional layer; to avoid areas of infarction, necrosis, and hemorrhage; and to calculate the average value. The MTR asymmetry (MTRasym) map at the offset of 3.5 ppm is called the APTw image: APTw = MTRasym (3.5 ppm) = MTR (3.5 ppm) – MTR (−3.5 ppm) = [Ssat (−3.5 ppm) – Ssat (3.5 ppm)]/S0.

### Statistical analysis

All variables were assessed for normality using the Shapiro–Wilk test. Data are presented as mean ± standard deviation (SD) or median and interquartile range for continuous data and frequency for categorical data. The independent sample *t*-tests were used to compare the difference between HCs and aMCI groups when the continuous data follow a normal distribution; the Mann–Whitney *U*-tests were used when continuous data are not normally distributed. An intraclass correlation coefficient (ICC) of 95% was used to evaluate the intrarater reliability regarding the measurements of APTw values. The correlation between Glx/GABA+ levels and APTw values was tested using Spearman's correlation coefficient. Binary logistic regression and receiver operating characteristic (ROC) curve analyses were used to evaluate the diagnostic performance of MRS parameters and APTw values. Statistical analyses were carried out by GraphPad Prism 7.0 and SPSS software (version 17.0), and statistical significance was determined by a *P*-value of < 0.05.

## Results

### Demographic characteristics

In Table 1, demographic and clinical data are presented. Age, gender, and educational level were not different between the HCs and aMCI groups (*P* > 0.05). The mean MMSE scores in the aMCI group were significantly lower than those in the HCs group (26.6 ± 0.31 vs. 23.7 ± 0.61, *P* < 0.001). The CDR value of each patient with aMCI was 0.5 and of each healthy control was 0.

**Table 1:** Demographics and cognitive scores of all participants.

	**HCs (20)**	**aMCI (20)**	***P*-value**
Number	20	20	/
Age (years)	65.53 ± 1.13	63.13 ± 1.70	0.248^b^
^*^Gender (men/women)	8/12	9/11	0.749^a^
Education (years)	10.27 ± 0.88	9.47 ± 0.98	0.548^b^
MMSE	26.6 ± 0.31	23.7 ± 0.61	<0.001^b^
CDR	0.5	0	<0.001^b^

HCs, Heathy Controls; aMCI, Amnestic Mild Cognitive Impairment; MMSE, Minimum Mental State Examination; CDR, Clinical Dementia Rating; Unless otherwise indicated data are presented as mean ± standard deviation (SD);

* Data are presented as frequencies;

a Chi-square test;

b Independent Sample t-test.

### GABA+ and Glx levels of the right hippocampus

Fitting errors of GABA+ and Glx in all participants were <15% and did not differ between patients with aMCI and HCs (GABA+: 8.19 ± 0.38% vs. 8.61 ± 0.34%, *P* = 0.413; Glx: 8.85 ± 1.97% vs. 9.22 ± 1.55%, *P* = 0.546). All VOIs had linewidth below 15 Hz, and there was no significant difference between the two groups (*P* = 0.457). The mean linewidth was 12.03 Hz for the aMCI group and 11.49 Hz for the HCs group. The GM tissue fractions [GM/(GM + WM)] within the spectroscopic VOI between the groups revealed no differences (HCs vs. aMCI: 55.39 ± 3.90 % vs. 53.33 ± 4.17 %, *P* = 0.211). The detailed results are presented in Table 2.

**Table 2:** MRS quantification and voxel segmentation results.

	**HCs (20)**	**aMCI (20)**	*P*-value^*^
GABA+fitting error (%)	8.19 ± 0.38	8.61 ± 0.34	0.413
Glx fitting error (%)	8.85 ± 1.97	9.22 ± 1.55	0.546
Linewidth (Hz)	11.49 ± 0.57	12.03 ± 0.44	0.457
GM volume fraction (%)	50.20 ± 4.86	48.87 ± 4.03	0.382
WM volume fraction (%)	40.53 ± 5.22	42.80 ± 4.43	0.189
GM tissue fraction	55.39 ± 3.90	53.33 ± 4.17	0.211

HCs, Heathy Controls; aMCI, Amnestic Mild Cognitive Impairment; GABA, Gamma-aminobutyric Acid; Glx, Glutamate-glutamine; GM, Gray Matter, WM, White Matter; Data are presented as mean ± SD;

* Independent Sample t-test.

**Figure 3:**
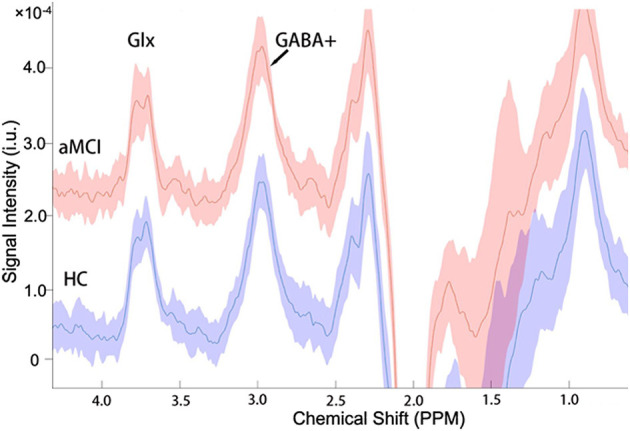
The mean (±standard deviation) GABA+ and Glx edited spectra acquired by the MEGA-PRESS sequence in the healthy controls (HCs) and patients with aMCI. The signal intensities of the aMCI group consistently plus a constant of 2.0 × 10^−4^ for more convenient signal display and comparison.

**Figure 4:**
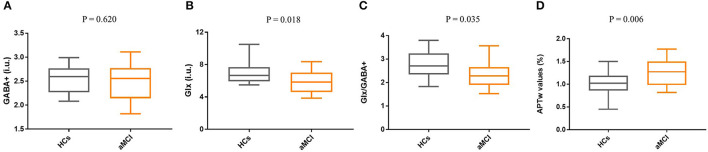
Box and whisker plots for the comparison of Glx levels, GABA+ levels, Glx/GABA+ ratios, and APTw values between the HCs group (*n* = 20) and the aMCI group (*n* = 20). **(A)** No significant difference in GABA+ levels was found between HCs and aMCI (*P* = 0.620). **(B)** Compared with the HCs group, Glx levels of the aMCI group were significantly decreased (*P* = 0.018). **(C)** The Glx/GABA+ ratios of the aMCI group are lower than the HCs, and a statistical difference was detected (*P* = 0.035). **(D)** Compared with HCs, patients with aMCI showed higher APTw values in the right hippocampus (*P* = 0.006).

The mean (± standard deviation) GABA+ and Glx edited spectra acquired by the MEGA-PRESS sequence for the healthy controls and patients with aMCI are shown in Figure 3. The Glx levels were significantly lower in the right hippocampus of patients with aMCI than those in HCs (7.02 ± 1.41 i.u. vs. 5.81 ± 1.33 i.u., *P* = 0.018, Figure 4A). However, no significant differences of GABA+ levels were observed in patients with aMCI (HCs vs. aMCI: 2.54 ± 0.28 i.u. vs. 2.47 ± 0.36, *P* = 0.620, Figure 4B). Compared with the HCs, patients with aMCI showed significantly lower Glx/GABA+ ratios (2.79 ± 0.60 vs. 2.37 ± 0.55, *P* = 0.035, Figure 4C). The detailed results are presented in Table 3.

**Table 3:** Difference of right hippocampal metabolites between patients with HCs and patients with aMCI.

	HCs (20)^*^	aMCI (20)^*^	***P*-value^#^**
GABA+ (i.u.)	2.60 (0.50)	2.56 (0.32)	0.620
Glx (i.u.)	6.67 (1.75)	5.84 (2.41)	0.018
Glx/GABA+ Ratios	2.71 (0.89)	2.29 (0.76)	0.035
APTw (%)	1.02 (0.33)	1.28 (0.52)	0.006

HCs, Heathy Controls; aMCI, Amnestic Mild Cognitive Impairment; GABA, Gamma-aminobutyric Acid; Glx, Glutamate-glutamine; APTw, Amide Proton Transfer-weighted;

* Data are presented as median (interquartile range);

# Mann–Whitney U-tests.

### Right hippocampal APT

The ICC of APT measurements was 0.961 (95% CI: 0.92–0.98). Compared with HCs, patients with aMCI demonstrated higher APTw values in the right hippocampus (0.99 ±0.26 % vs. 1.26 ± 0.28%, *P* = 0.006, Figure 4D).

### Correlation analysis of right hippocampus metabolites

**Figure 5:**
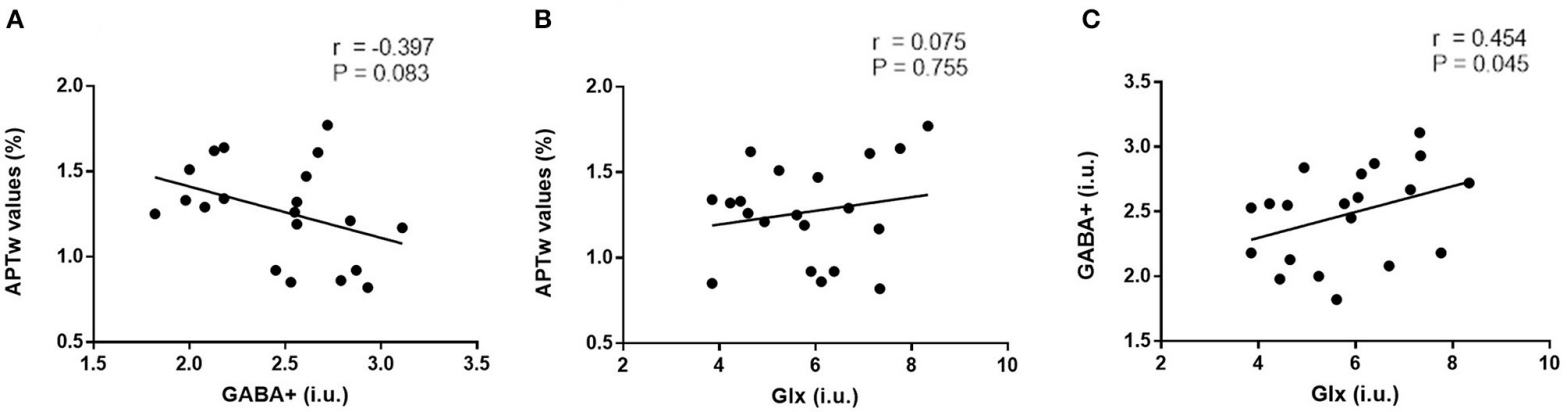
Correlation analysis of right hippocampus metabolites. No significant correlation was found between APTw values and GABA+ levels of the right hippocampus [*r* = −0.39, *P* = 0.083, **(A)**]; similar results were found between APTw values and Glx levels [*r* = 0.074, *P* = 0.755, **(B)**]. There is a moderate positive correlation between Glx and GABA+ levels [*r* = 0.454, *P* = 0.045, **(C)**].

No significant correlation was found between APTw values and GABA+ levels of the right hippocampus (*r* = −0.39, *P* = 0.083, Figure 5A); similar results were found between APTw and Glx values and levels (*r* = 0.074, *P* = 0.755, Figure 5B). There is a moderate positive correlation between Glx and GABA+ (*r* = 0.454, *P* = 0.045, Figure 5C).

### Diagnostic capability of right hippocampus metabolites for aMCI

**Figure 6:**
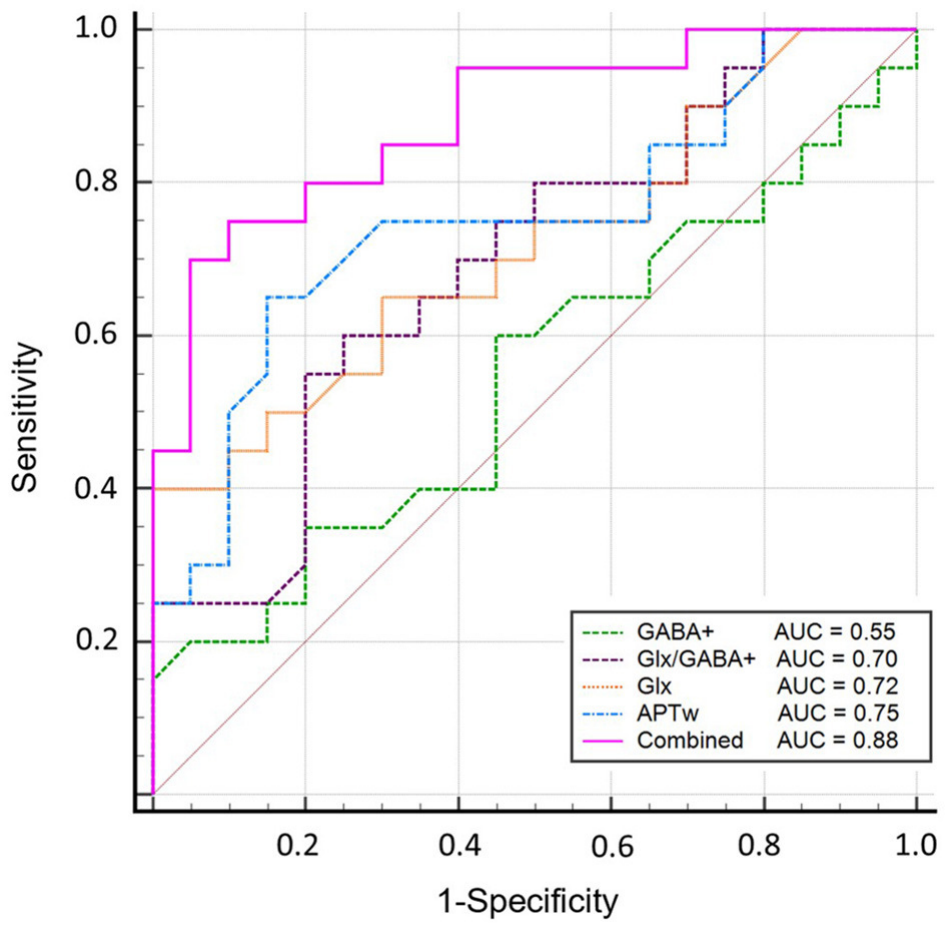
Diagnostic performance of Glx, GABA+, Glx/GABA+ ratios, and APTw and the combined parameters. The ROC curve analysis showed that GABA+ has the lowest AUC of 0.55; Glx, Glx/GABA+ ratios, and APTw values have similar areas under the curve (AUC) of 0.72, 0.70, and 0.75, respectively, for the diagnosis of aMCI; a combination of these parameters demonstrated an increased AUC value of 0.88.

Glutamate–glutamine (Glx) yielded 100% of specificity and positive predictive value (PPV) but the lowest sensitivity (40%) for diagnosing aMCI. In addition, GABA+ had lower sensitivity (60%), specificity (55%), and accuracy (58%). The sensitivity, specificity, accuracy, PPV, and negative predictive value (NPV) were 65, 85, 75, 81, and 71%, respectively. The ROC curve analysis showed that Glx, GABA+, Glx/GABA+ ratios, and APTw values had an area under the curve (AUC) of 0.72, 0.55, 0.70, and 0.75, respectively, for the diagnosis of aMCI. These variables were introduced into the logistic regression analysis, and a combination of the independent parameters yielded an increased AUC of 0.88 for its diagnostic performance, which is higher than univariate analysis (*P* < 0.05). In the regression model, β of APT and Glx was 5.94 and−2.78, respectively. Detailed comparison results are shown in Supplementary Table 1. The sensitivity and specificity of the combined markers were 0.7 and 0.95, respectively. The ROC curves are shown in Figure 6. The detailed diagnostic capability of univariate and multivariate variables is shown in Table 4.

**Table 4:** Diagnostic performance of right hippocampal metabolites.

	**Sensitivity**	**Specificity**	**Accuracy**	**PPV**	**NPV**	**LR+**	**LR-**	**AUC**	***P*-value^#^**
GABA+	0.60	0.55	0.58	0.60	0.58	1.33	0.73	0.55	0.620
Glx	0.40	1.0	0.70	1.0	0.63	/	0.60	0.72	0.018
Glx/GABA+ Ratios	0.60	0.75	0.68	0.71	0.65	2.40	0.53	0.70	0.035
APTw	0.65	0.85	0.75	0.81	0.71	4.33	0.41	0.75	0.006
Combined	0.70	0.95	0.83	0.93	0.76	14.0	0.32	0.88	< 0.001

GABA, Gamma-aminobutyric Acid; Glx, Glutamate-glutamine; APTw, Amide Proton Transfer-weighted; PPV, Positive Predictive Value; NPV, Negative Predictive Value; LR, Likelihood Ratio; AUC, Area Under Curve;

# Mann–Whitney U-tests.

## Discussion

In the present study, we preliminary explored the changes of Glx/GABA+ levels and APTw in the hippocampus in patients with aMCI using MEGA-PRESS and APTw imaging. Patients with aMCI exhibited decreased Glx levels (and Glx/GABA+ ratios) and increased APTw values in the right hippocampus. The combination of Glx and APTw values improved the diagnostic performance for aMCI, suggesting it as a potential imaging diagnostic marker.

Only a few researchers focused on hippocampal Glx levels in patients with MCI (Rupsingh et al., 2011; Huang et al., 2017; Wong et al., 2020) with diverse results: one 7T MRS study with 33 participants demonstrated decreased left hippocampal Glu in patients with MCI (Wong et al., 2020), a 3T MRS study with 21 MCI patients showed a decreasing trend of Glx without significant difference in the right hippocampus (Huang et al., 2017), and finally, a 4T MRS study with 12 MCI showed no obvious changes of Glu in the right hippocampus (Rupsingh et al., 2011). Our study showed that patients with aMCI exhibit lower Glx levels and Glx/GABA+ ratios in the right hippocampus than in HCs, reflecting a potential hippocampal excitatory and inhibitory imbalance in patients with aMCI. However, in this research, no significant changes in GABA+ levels were observed in patients with aMCI, consistent with Huang et al. (2017) study. According to previous studies, glutamatergic neurotransmission in the hippocampus is severely disrupted in AD (Butterfield and Pocernich, 2003) and is predominantly affected in the early stages of AD (Canas et al., 2014). Moreover, a mouse model study showed significantly decreased Glu levels in mice with AD but no significant changes in GABA levels (Nilsen et al., 2012). Therefore, the changes in glutamatergic neurons may precede GABAergic neurons in E/I systems in the process of AD and altered Glx level may be a significant marker for early diagnosis of MCI.

Increased APTw values were found in patients with aMCI, consistent with prior studies based on APTw imaging (Zhang et al., 2016, 2020). APTw sequence is sensitive to mobile proteins having a specific amide proton resonance at the frequency of 3.5 ppm downfield from water. Theoretically, an increasing concentration of amide protons should result in a higher APTw value. Abnormal protein accumulation, such as extracellular amyloid plaques and intracellular neurofibrillary tangles, is a crucial pathological change of the MCI brain, and the accumulation of these abnormal proteins might contribute to the increase of APTw signals. Therefore, APTw would be a promising imaging marker for the diagnosis of MCI.

Glx/GABA+ ratios and APTw values had a moderate diagnostic capability for aMCI, but GABA+ had a poor diagnostic performance. Glx yielded 100% specificity and PPV but the lowest sensitivity (40%) for diagnosing aMCI, which limited its independent clinical application. No significant correlation was found between Glx levels and APTw values, indicating a promising complementary role between the two parameters for the diagnosis of aMCI. In the regression model, APT and Glx had a strong correlation with aMCI. A combination of these parameters demonstrated an increased AUC and improved diagnostic performance, suggesting a potential imaging diagnostic strategy.

To the best of our knowledge, the heterogeneity of the B0 magnetic field, large susceptibility gradients, and poor signal-to-noise ratio in the hippocampal region are the major challenges in acquiring the edited MRS. In our study, a 60 cm bore 3.0 tesla MRI scanner provided a highly homogeneous magnetic field for MRS. In addition, the VOI was positioned parallel to the long axis of the hippocampal body and covered the maximum of the hippocampus avoiding the bone and the sinuses. The linewidth for all acquired spectra was below 15 Hz. In the data processing, all spectra underwent frequency alignment for motion correction. With these efforts, high-quality spectra were obtained in the right hippocampus at 3 T.

In our study, patients with aMCI were recruited from a neurology outpatient clinic in Jinan, and healthy participants were from the communities in Jinan. There may be a sample bias between the two groups. To reduce possible bias, every participant was recruited in accordance with the same detailed screening process. Moreover, there is no significant difference in demographics between patients and healthy participants, which may decrease the possible bias.

There are some limitations of our study. First, our preliminary study was based on a small sample. Although only 20 aMCI and 20 HCs were recruited, the results demonstrated the feasibility of the combined imaging diagnostic marker. In future, our goal is to recruit more participants and carry out large-scaled research to verify and enhance the results. Second, Glu and Gln signals were not separated, but considered collectively as Glx, which is more likely a marker of neuronal metabolism than neurotransmission *per se* (Song et al., 2022). Third, the MEGA-PRESS method detects GABA+ signals containing significant contributions (~50%) from macromolecules (MMs). New methods to isolate GABA in the spectrum are desirable to detect even tiny changes in GABA. Fourth, the freehand ROI of APTw imaging was not identical to the VOI of MRS. ROIs were drawn in the maximum cross-sectional layer of the right hippocampus to avoid being contained to the surrounding brain structure, diminishing the possible errors.

## Conclusion

Patients with aMCI exhibited decreased Glx levels and Glx/GABA+ ratios and increased APTw values in the right hippocampus. A combination of Glx levels and APTw values improved the diagnostic performance for aMCI, suggesting it as a promising combined imaging diagnostic marker. Although this is a preliminary exploration based on a small sample, the results demonstrated the feasibility of the combined imaging diagnostic marker. The potential imaging diagnostic strategy of aMCI may promote the early detection of aMCI and facilitate timely intervention to delay the pathological progress toward AD.

## Data availability statement

The raw data supporting the conclusions of this article will be made available by the authors, without undue reservation.

## Ethics statement

The studies involving human participants were reviewed and approved by Institutional Review Board of Shandong Provincial Hospital. The patients/participants provided their written informed consent to participate in this study.

## Author contributions

XC: conceptualization, data curation, methodology, writing original draft, and funding acquisition. TG: conceptualization, resources, methodology, and writing – review and editing. TC: resources and data curation. CX: data curation. YL: writing – review and editing. QS: statistical analysis. LL: methodology, statistical analysis, and writing – review and editing. GO: methodology. RE: methodology and writing – review and editing. GW and ZX: supervision, study design, project administration, writing – review and editing, and funding acquisition. All authors contributed to the article and approved the submitted version.
