# Supplementary figures and images for: Altered glutamate–glutamine and amide proton transfer-weighted values in the hippocampus of patients with amnestic mild cognitive impairment: A novel combined imaging diagnostic marker
Source: Front Neurosci. 2023 Feb 23;17:1089300. doi: 10.3389/fnins.2023.1089300 (PMC9995585; doi:10.3389/fnins.2023.1089300)

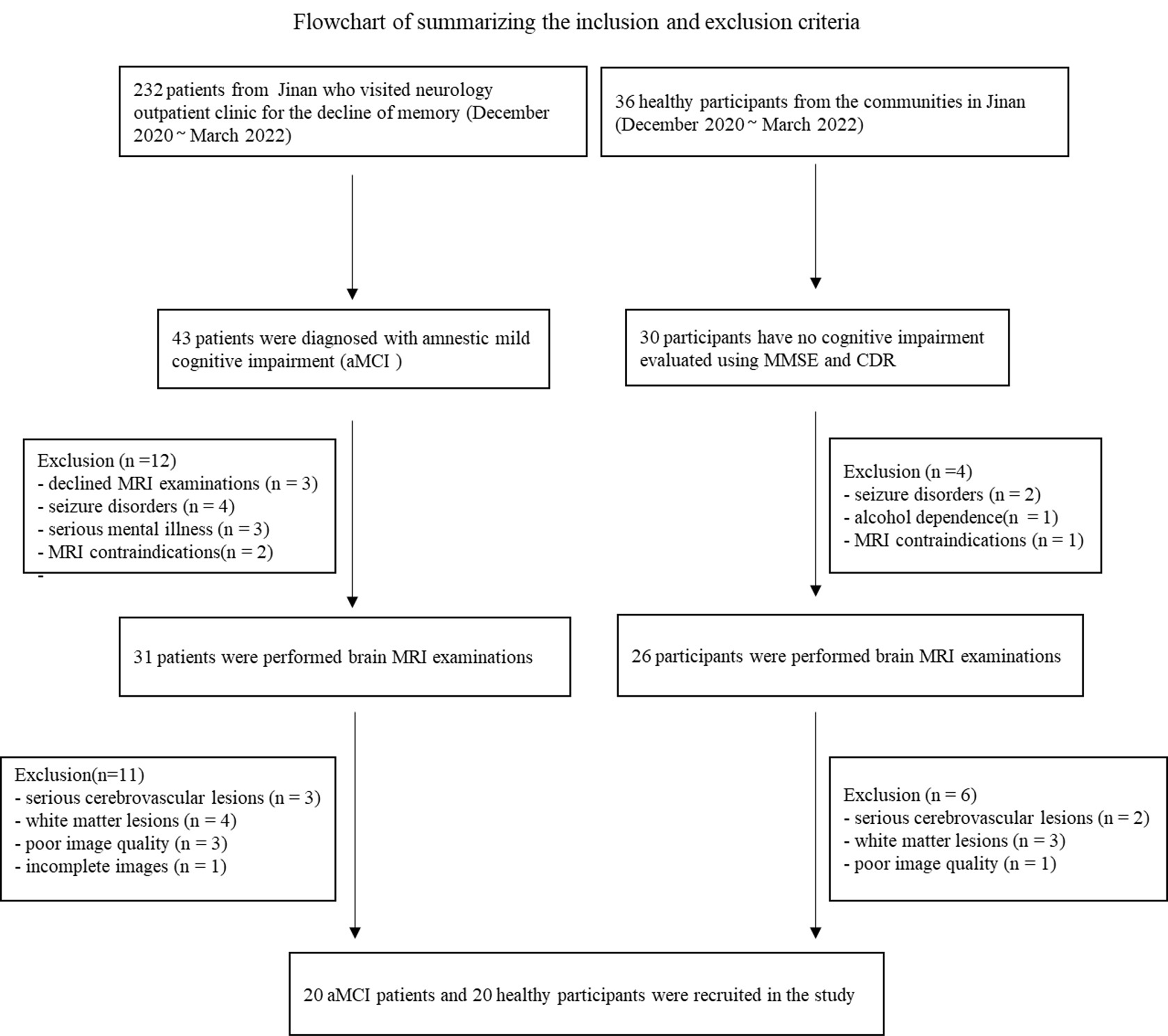

Supplement: Supplementary file 1 [file Image_1.JPEG]
