# Supplementary material for: Altered glutamate–glutamine and amide proton transfer-weighted values in the hippocampus of patients with amnestic mild cognitive impairment: A novel combined imaging diagnostic marker
Source: Front Neurosci. 2023 Feb 23;17:1089300. doi: 10.3389/fnins.2023.1089300 (PMC9995585; doi:10.3389/fnins.2023.1089300)
Supplement: Supplementary file 2 [file Table_1.DOCX]

Detailed comparation between univariate analysis and combined multivariate analysis

| APT ~ Combined | |
| --- | --- |
| Difference between areas | 0.132 |
| Standard Error | 0.0642 |
| 95% Confidence Interval | 0.00661 to 0.258 |
| z statistic | 2.063 |
| Significance level | P = 0.0391 |
| GABA ~ Combined | |
| Difference between areas | 0.335 |
| Standard Error | 0.0987 |
| 95% Confidence Interval | 0.142 to 0.528 |
| z statistic | 3.395 |
| Significance level | P = 0.0007 |
| Glx ~ Combined | |
| Difference between areas | 0.166 |
| Standard Error | 0.0761 |
| 95% Confidence Interval | 0.0172 to 0.315 |
| z statistic | 2.186 |
| Significance level | P = 0.0288 |
| Glx/GABA+ Ratio ~ Combined | |
| Difference between areas | 0.186 |
| Standard Error | 0.0817 |
| 95% Confidence Interval | 0.0262 to 0.346 |
| z statistic | 2.281 |
| Significance level | P = 0.0226 |
