# Supplementary material for: Altered glutamate–glutamine and amide proton transfer-weighted values in the hippocampus of patients with amnestic mild cognitive impairment: A novel combined imaging diagnostic marker
Source: Front Neurosci. 2023 Feb 23;17:1089300. doi: 10.3389/fnins.2023.1089300 (PMC9995585; doi:10.3389/fnins.2023.1089300)
Supplement: Supplementary file 3 [file Data_Sheet_1.docx]

**The inclusion criteria for amnestic mild cognitive impairment(aMCI):**

MCI that with **memory impairments** was classified into aMCI. Core clinical criteria for the diagnosis of MCI were as follows: **1. an abnormal change in cognition**: obtained from the patient, from an informant who knows the patient well, or from the neurologist observing the patient. **2. Impairment in one or more cognitive domains** (obtained from cognitive testing): lower performance in one or more cognitive domains that is greater than would be expected for the patient’s age and educational background. **3. Preservation of independence in functional abilities:** perform complex functional tasks with mild problems, maintain their independence of function in daily life with minimal aids or assistance. **4. Not demented**: cognitive changes should be sufficiently mild that there is no evidence of a significant impairment in social or occupational functioning.

Reference:

*1. Petersen RC. Clinical practice. Mild cognitive impairment. N Engl J Med. 2011;364(23):2227-2234.*

*2. Albert MS, DeKosky ST, Dickson D, et al. The diagnosis of mild cognitive impairment due to Alzheimer's disease: recommendations from the National Institute on Aging-Alzheimer's Association workgroups on diagnostic guidelines for Alzheimer's disease. Alzheimers Dement. 2011;7(3):270-279.*
